# Exosomal Composition, Biogenesis and Profiling Using Point-of-Care Diagnostics—Implications for Cardiovascular Disease

**DOI:** 10.3389/fcell.2022.853451

**Published:** 2022-06-01

**Authors:** Denise Burtenshaw, Brian Regan, Kathryn Owen, David Collins, David McEneaney, Ian L. Megson, Eileen M. Redmond, Paul Aidan Cahill

**Affiliations:** ^1^ Vascular Biology and Therapeutics, School of Biotechnology, Dublin City University, Dublin, Ireland; ^2^ School of Biotechnology, Dublin City University, Dublin, Ireland; ^3^ Southern Health and Social Care Trust, Craigavon Area Hospital, Craigavon, United Kingdom; ^4^ Nanotechnology and Integrated Bioengineering Centre (NIBEC), Ulster University, Belfast, United Kingdom; ^5^ Division of Biomedical Sciences, Centre for Health Science, UHI Institute of Health Research and Innovation, Inverness, United Kingdom; ^6^ Department of Surgery, University of Rochester, Rochester, NY, United States

**Keywords:** exosome (vesicle), atheroscelorsis, stem cell repair mechanisms, endothelial (dys)function, point of care diagnosis

## Abstract

Arteriosclerosis is an important age-dependent disease that encompasses atherosclerosis, in-stent restenosis (ISR), pulmonary hypertension, autologous bypass grafting and transplant arteriosclerosis. Endothelial dysfunction and the proliferation of vascular smooth muscle cell (vSMC)-like cells is a critical event in the pathology of arteriosclerotic disease leading to intimal-medial thickening (IMT), lipid retention and vessel remodelling. An important aspect in guiding clinical decision-making is the detection of biomarkers of subclinical arteriosclerosis and early cardiovascular risk. Crucially, relevant biomarkers need to be good indicators of injury which change in their circulating concentrations or structure, signalling functional disturbances. Extracellular vesicles (EVs) are nanosized membraneous vesicles secreted by cells that contain numerous bioactive molecules and act as a means of intercellular communication between different cell populations to maintain tissue homeostasis, gene regulation in recipient cells and the adaptive response to stress. This review will focus on the emerging field of EV research in cardiovascular disease (CVD) and discuss how key EV signatures in liquid biopsies may act as early pathological indicators of adaptive lesion formation and arteriosclerotic disease progression. EV profiling has the potential to provide important clinical information to complement current cardiovascular diagnostic platforms that indicate or predict myocardial injury. Finally, the development of fitting devices to enable rapid and/or high-throughput exosomal analysis that require adapted processing procedures will be evaluated.

## Introduction

Human arteriosclerotic disease is a complex systemic inflammatory disorder characterized by key interactions between several different biological modifiers. These include various lipids, enzymes, endothelial cells (ECs), vascular smooth muscle cells (vSMCs), cytokines and both circulating and resident vascular stem cells and mononuclear cells (Bennett et al., 2016). Initial endothelial dysfunction leads to intimal medial thickening (IMT) and lipid retention resulting in inflammatory and fibroproliferative responses that culminate in cellular infiltration, deposition of extracellular matrix (ECM), and the formation of atherosclerotic plaques. Consequently, unstable atherosclerotic plaques can lead to MI or ischaemic stroke.

Numerous pathologic observations suggest that early adaptive “transitional” lesions enriched with vSMC-like cells are commonly associated with atherosclerotic-prone regions of arteries. These adaptive lesions have been identified prior to pathologic intimal thickening, lipid accumulation and the presence of a developed plaque (Nakagawa and Nakashima, 2018). Similarly, endothelial dysfunction due to aberrant blood flow is associated with adaptive atheroprone lesion formation (Halcox et al., 2009), while the embryological origin of the arterial SMCs within the vessel wall may dictate lesion localisation and progression (Cheung et al., 2012). The progression of these adaptive lesions is an important therapeutic target and can be modelled *in vivo* using wildtype mice following flow restriction due to carotid artery ligation (Korshunov and Berk, 2003) and can further progress into advanced lesions using *ApoE* gene-deficient mice concurrently fed on a western diet (Nam et al., 2009; Liu et al., 2011).

It is widely recognised that vSMC-like cells are primarily responsible for the majority of neointimal cells in arteriosclerotic lesions following vessel injury in murine models [balloon angioplasty, coronary artery by-pass grafting (CABG), transplant arteriosclerosis, pulmonary hypertension and in-stent restenosis (ISR)] (Bennett et al., 2016). This vascular fibrosis and IMT is characterized by a reduced lumen diameter due to excessive deposition of ECM, inhibition of matrix degradation and the accumulation of neointimal cells (mostly vSMC-like cells and macrophages) (Lan et al., 2013).

Sonographic measurement of carotid intima-media thickness (cIMT) is widely used as an indicator of not only local, but generalised atherosclerosis, even in asymptomatic individuals (Touboul et al., 2005). Indeed, cIMT is not only an important surrogate marker for disease synonymous with subclinical atherosclerosis, but can also result from non-atherosclerotic processes such as vascular ageing (Qu and Qu, 2015), whereby an increase in IMT is observed even in populations with a low incidence of lipid driven atherosclerosis (Al Rifai et al., 2018). IMT increases threefold between ages 20–90 years and may at a given age predict future outcomes that can be accelerated in the presence of known CVD risk factors such as Low density lipoprotein (LDL). Hence, IMT typical of adaptive lesions in early subclinical atherosclerosis provides an important substrate for lipoprotein retention leading to accelerated atherosclerotic plaque formation and acute coronary syndrome (ACS) (Nakashima et al., 2008). When combined with elevated fasting remnant cholesterol levels, cIMT has also recently been successfully deployed in stratifying patients with ischemic stroke and optimal LDL cholesterol levels (Qian et al., 2021). Thus, sonographic detection and evaluation of atherosclerosis through cIMT and more recently, femoral plaque assessment, is fast-becoming critical to clinical decision-making in diagnostic evaluation of at-risk and symptomatic atherosclerotic patients.

Pre 2015 studies reveal little standardisation relating to the methods used for cIMT measurement, which gave rise to a disparity in the ability of cIMT to predict risk classification (van den Oord et al., 2013). From 2015 onwards, an industry-standard method was commonly adopted, with the measurement acquired at a plaque-free site >0.5 mm at the distal end near the carotid bifurcation (Ûygarden, 2017). Since this methodology became established results, have consistently found cIMT to be a reliable biomarker for CAD. More recently, several extensive studies have considered the inclusion of plaque measurements, which proved to be a helpful addition to traditional risk factors, giving the ability to predominantly reclassify the intermediate-risk patient group (Amato et al., 2017). However, as with the early cIMT studies, a defined methodology needs to be established, as plaque measurement techniques have been inconsistent.

Chronic coronary artery disease typical of AMI is usually associated with a rupture of atherosclerotic plaque, thrombus formation and obstruction of blood flow leading to necrosis of myocardium. However, in some patients with AMI, there are no significant lesions present in coronary arteries interrogated by angiography, thus confounding early identification of atherosclerosis. ST-segment elevation myocardial infarction (STEMI), non-ST-segment elevation myocardial infarction (NSTEMI), unstable angina and stable coronary artery disease (CAD) all carry significant morbidity and mortality (Dong et al., 2015). As a result, prompt diagnosis and appropriate treatment is essential in preventing adverse outcomes. Electrocardiogram (ECG) analysis and morphological features suggest STEMI have smaller lumen areas, greater plaque burden, and are more prone to plaque rupture compared with lesions causing NSTEMI/unstable angina or stable CAD (Dong et al., 2015).

The origin of the neointimal cells that cause IMT has attracted much debate and controversy with numerous animal studies raising two major possibilities for their proliferation: *1*) partial somatic cell reprogramming following de-differentiation (and in some cases within calcified lesions, trans-differentiation) of mature differentiated vSMCs or endothelial-mesenchymal transition (EndoMT) of ECs to vSMC-like cells (Nemenoff et al., 2011; Chappell et al., 2016; Cooley et al., 2014; Evrard et al., 2017), *2*) partial differentiation of immature multipotent “mesenchymal-like” vascular stem cells (MVSCs) that reside within the vessel wall or migrate from the circulation (Tang et al., 2012; Tang et al., 2020; Kramann et al., 2016; Wan et al., 2012). Several initial studies have provided important evidence for the involvement of a discrete subpopulation of differentiated medial SMCs in progressing IMT (Chappell et al., 2016; Dobnikar et al., 2018). However, lineage tracing analysis studies have also implicated glioma-associated oncogene-1 (Gli1) adventitial progenitors (Baker and Péault, 2016) SRY-related HMG-box 10 (Sox10) and S100β adventitial and medial vascular stem cells (Yuan et al., 2017; Di Luca et al., 2021; Molony et al., 2021), in addition to Nestin MSC-like cells (Wan et al., 2012), in contributing to vascular fibrosis and the generation of intimal lesions following injury. Importantly, recent studies also suggest that some of these adventitial progenitors may be derived from a parent Myh11 SMC population (Majesky et al., 2017).

Myocardial infarction with non-obstructive coronary arteries (MINOCA) also involves a heterogenous group of atherosclerotic and non-atherosclerotic patients resulting in myocardial damage that is not due to obstructive coronary artery disease (Singh et al., 2021). While there are many sources of neointimal vSMC-like cells, in order to truly elaborate on the pathological events that occur during the onset of SMC de-differentiation, phenotypic modulation and/or stem cell differentiation, an understanding of the pivotal role of the vascular endothelium as the primary responder to injury in dictating disease progression is essential. Composed of a single layer of cells, the endothelium acts as the front line of defence by maintaining homeostasis and the release of anti-atherogenic protective molecules in addition to protecting the internal layers of the artery from circulating atherogenic molecules in the bloodstream. It is widely accepted that the endothelium is the primary responder to known risk factors associated with CVD, including diabetes, high-fat diets, physical inactivity along with several other metabolic disorders. Endothelial dysfunction (ED) marks the initiation of a cascade of events of primary importance to the pathogenesis of vascular disease (Cahill and Redmond, 2016).

## Extracellular Vesicles, the Cellular Postmen!

Initially thought to be merely a disposal mechanism for cellular waste, extracellular vesicles (EVs) have recently become a focal point of research due to their multifunctional role in many biological processes, including cell-cell communication (Bang and Thum, 2012), protein clearance (Johnstone et al., 1987), regulation of immune response (Bobrie et al., 2011), cancer (Muralidharan-Chari et al., 2010) and development of many vascular disease (Jansen et al., 2017). Various biological mechanisms constitutively release these heterogeneous population of phospholipid bilayer-enclosed EVs from discrete cell types including, immune cells, tumour cells, neurons, and ECs. The molecular constituents found within circulating EVs include a variety of proteins, DNA and microRNAs (miRNAs). These are released into the extracellular environment where they are captured and internalised by neighbouring cells. Alternatively, they can potentially travel through the systemic circulation to access neighbouring tissues. This unique mode of intercellular communication between cells and tissues within the body facilitated by EVs constitutes their potential use as novel pathological circulating biomarkers in the discovery of and development of clinical diagnostic tools.

### Characterisation of Extracellular Vesicles

#### Apoptotic Bodies

EVs are released in various shapes and forms by mammalian and non-mammalian cells. Due to their small size, differentiation between EVs of different origin proves challenging; however, they can be characterised to some extent by their size, content, and sub-cellular origin (Colombo et al., 2014). There are three main mechanisms by which extracellular vesicles may arise, resulting in three distinct subpopulations of vesicles (van der Pol et al., 2012). The first and most widely studied involves cell fragmentation during a programmed cell death process known as apoptosis. During this process, the cell undergoes several morphological changes resulting in membrane blebbing, membrane protrusion and subsequent release of apoptotic bodies. Apoptotic bodies are the largest sub-population of EVs and range between 1 and 5 µm in diameter (Kerr et al., 1972). Additionally, they consist of membrane-bound cytoplasm with tightly packed organelle and nuclear debris from the host cell. Once secreted into the extracellular space, these vesicles undergo phagocytosis by neighbouring immune cells (Arandjelovic and Ravichandran, 2015).

#### Microvesicles

The second method of EV generation involves the shedding of membrane-bound sacs, known as microvesicles, from various cell types. During this process, small cytoplasmic vesicles protrude from the host cell membrane, which then detaches through a blebbing process. The mechanism that drives this process is not fully understood; however, the rate of the shedding process is known to be dramatically increased by a rise in cytosolic concentrations of calcium ions. The increase of calcium ions activates scramblase and calpain, resulting in a loss of membrane phospholipid symmetry and degradation of various proteins, thus facilitating outward budding of microvesicles from the cellular membrane (Yuana et al., 2013). These EVs can vary widely in size, but generally fall between 100 nm and 1 µm in diameter. The cargo packaged within these cells includes proteins, DNA, and microRNA’s (miR’s); however, not all microvesicles will carry the same messages. The proteins carried within are indicative of the host cell from which they have departed (Del Conde et al., 2005).

#### Exosomes

As the smallest subset of EVs, exosomes range between 30 and 100 nm in size. These endosomal derived microvesicles first came to light in the 1980s when researchers Pan, Stahl, and Johnstone reported their existence in the extracellular space while studying the maturation of reticulocytes to erythrocytes *via* exosomal secretion of transferrin receptors in the bloodstream (Harding and Stahl, 1983). These vesicles are double-layered, with the lipid bilayer corresponding to that of the cells from which they are released. The release of exosomes from cells was initially considered to cellular waste secreted as a function of cellular homeostasis and bearing no significant impact on neighbouring cells and tissues.

Interestingly, however, in the past decade, it has been widely accepted that these vesicles are functional vehicles carrying a variety of membrane-bound complex cargo of lipids (Vidal et al., 1989), proteins (Simpson et al., 2009), DNA, mRNA and miR’s (Valadi et al., 2007). This unique process enables the delivery of these cargos to proximal and distal cells and tissues initiating a response, thus representing a mode of intercellular communication. Although initial discoveries indicated the release of exosomes from blood cells, the process has since been recognised to be a feature of almost all eukaryotic cells within the body including, but not limited to, various immune cells (dendritic cells, T-cells, B-cells, astrocytes) (Peters et al., 1989; Zitvogel et al., 1998) tumour cells (Verma et al., 2015), epithelial and resident vascular cells (cardiomyocytes, vSMCs, vascular progenitor cells, ECs) (Hergenreider et al., 2012; Zhao et al., 2017). Moreover, it is well established that their cargo may differ significantly and be dependent on the cell source. With this in mind, exosomes and their cargo may offer a potential insight into cell-cell communication as well as a prognostic information in various diseases such as cancers [94], neurodegenerative diseases [95], chronic inflammation [96], renal and CVD [97, 98].

### Exosome Biogenesis

Upon their discovery in the 1980s, the production of exosomes was reported to be *via* an intracellular process in the endosomal compartment of the cell. This process is initiated by an inward membrane budding of an early endosome into the luminal compartment, leading to the formation of multivesicular bodies (MVBs). Following this, invagination of late endosomal membranes results in the formation of intraluminal vesicles (ILVs) within large MVB’s (Pan et al., 1985; Johnstone et al., 1987).

It is at this stage that various proteins along with cytosolic compounds from the host cell are incorporated into the invaginating membrane-bound ILVs. There are two distinct pathways in which MVBs are generated—*1*) fusion to the plasma membrane and *2*) fusion with lysosomes and the degradation and recycling of their content (Babst, 2011).

### The Endosomal Sorting Complex Required for Transport-Dependent Pathway

**FIGURE 1:**
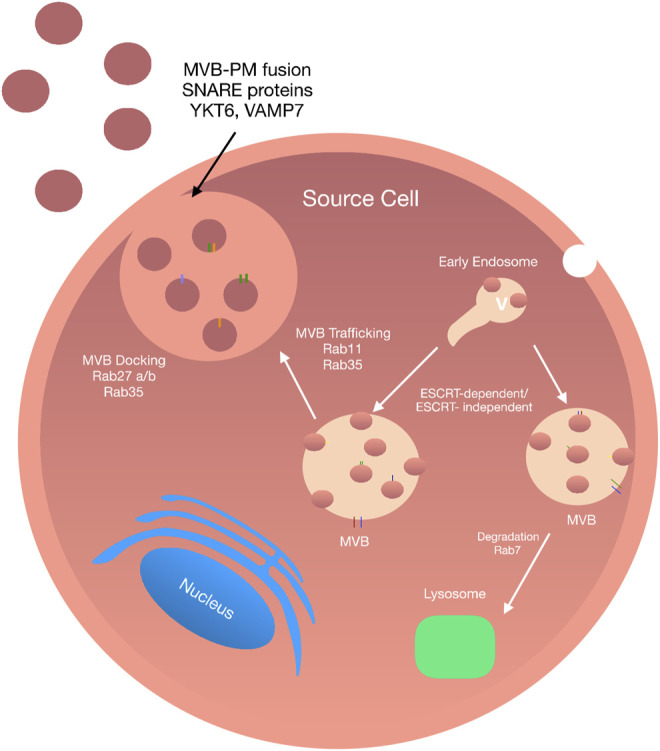
Exosome Biogenesis: Exosomes biogenesis is a complex process and can be conducted in an ESCRT-dependent and -independent manner. The internalisation of the plasma membrane initiates the formation of exosomes by endocytosis. The fusion of early endosomes results in the production of multivesicular bodies. Upon maturation, MVBs are directed to the plasma membrane *via* a tightly Rab GTPase dependent multistep process where they undergo a SNARE-mediated plasma membrane fusion event followed by secretion into the extracellular space.

There are currently several proposed models associated with the mechanism by which exosomes are formed in MVBs (Fabrikant et al., 2009; Falguières et al., 2009; Hurley and Hanson, 2010). However, the most robust system for eukaryotic cells is the utilization of the established endosomal sorting complex required for transport (ESCRT) system and possibly additional ESCRT-independent pathways (Colombo et al., 2013) (Figure 1). ILV formation requires integration of two distinct pathways. The first involves endosomal enrichment for tetraspanins CD9 and CD63, along with membrane reorganization (Pols and Klumperman, 2009), while the second involves recruitment of the ESCRT complex to the site of ILV formation. The ESCRT pathway consists of approximately 20 proteins sorted into four soluble multi-protein complexes (ESCRTs 0, I, II, and III) that work together to facilitate MVB formation, vesicle budding, and protein cargo sorting (Christ et al., 2017) through ubiquitin-binding subunits. Initiation of the pathway begins with recognition and sequential binding of ESCRT-0 to ubiquitylated proteins in the endosomal membrane (Meister et al., 2017). ESCRT-I complex is then recruited to the cytosolic side of the early endosomes by way of various stimuli, including tsg101, phosphatidylinositol 3-phosphate (PIP3) and hepatocyte growth factor-regulated tyrosine kinase substrate (HRS), which then activates the ESCRT-II (Fernandez-Borja et al., 1999; Bache et al., 2003; Tamai et al., 2010) and initiates the oligomerization and formation of the ESCRT-III complex to promote the budding processes before budding formation and cleavage. The ESCRT-111 complex then separates from the MVB membrane *via* the sorting protein Vps4 and is disassembled by AAA-ATPase suppressor-of-potassium-transport-growth-defect-1 protein (SKD1) (Liégeois et al., 2006). In summary, following endosomal membrane reorganization, ESCRT-I and -II initiate and drive intraluminal budding of the MVB, while ESCRT-III completes the budding and cleavage process resulting in the formation of ILVs ready for secretion.

### Endosomal Sorting Complex Required for Transport-Independent Pathway

Exosomes can also be formed in an ESCRT-independent manner. There are several ESCRT-independent mechanisms which demonstrate the successful generation of ILVs in MVBs following complete disruption of ESCRT function (Stuffers et al., 2009). Although ESCRT-independent biogenesis of exosomes does not disrupt the general formation of ILVs, the formation of specific ILV populations may be hindered (Edgar et al., 2014). The discovery of a ceramide-based mechanism of ILV biogenesis initiated the hypothesis that exosome formation was not ESCRT dependent. Trajkovic et al. (2008) have shown that exosome biogenesis requires the generation of ceramide, a cone-shaped lipid produced during the hydrolysis of sphingomyelin by the enzyme sphingomyelinase. Production of ceramide may then facilitate the generation of membrane domains by membrane invagination due to its cone-like structure and further induce ILV curvature. Further studies have shown that MVBs loaded with CD63-positive ILVs were formed following depletion of components essential for ESCRT complexes. In addition to this, several other ESCRT-independent generation of ILVs have been reported that involve tetraspanin-mediated organization of cellular proteins for endosomal sorting and release (Escola et al., 1998). Interestingly, it is possible that different pathways may act together, or even in parallel to promote ILV formation and release. This hypothesis was proposed by Baietti et al. (2012) during the discovery of an alternative ESCRT pathway involving the syndecan syntenin-ALIX.

The leading players facilitating vesicle formation, loading of proteins and exosome biogenesis include heparinase, syndecan heparan sulfate proteoglycans, phospholipase D_2_, ADP ribosylation factor 6, and syntenin. The formation of ILVs is dictated by the association of syntenin with ALIX and relies on the bioavailability of heparan sulfate, syndecans, ALIX, and ESCRTs (Baietti et al., 2012). A further study carried out by Hoshiono et al. (2013) investigating the growth of cancer cells, reported that ESCRTs and function in unisonwith ceramide to facilitate the formation of ILVs. To date, proteomic analysis of EVs suggests that a cell type can release mixed vesicles populations at any given time (Kowal et al., 2016). Therefore, it is not surprising that exosomal biogenesis may involve both ESCRT-dependent and independent pathways. However, despite the predominance of the ESCRT pathway as a primary driver of exosome biogenesis, a major challenge for the future is to gain a further understanding into how different regulators of exosome biogenesis interact with one another and the effect of each mediator on the production of diverse ILV populations.

### Exosome Structure and the Cargo Within

Since their discovery in the 1980s, exosomes have been regarded to be a miniature version of the host cell in which they originate. This concept predominantly originated from the complex nature of exosomes in terms of structure and content which is highly dependent on that of the parent cell. In general, exosomes are heavily loaded with a diverse range of bioactive materials such as proteins, lipids, and nucleic acids. In recent years extensive research has been carried out in a bid to identify and characterise exosomal content. This has led to the establishment of three main publicly accessible databases: EVpedia, Vesiclepedia, and Exocarta which include useful information such as protein, lipid and nucleic acid content, as well as isolation and purification procedures (Mathivanan and Simpson, 2009; Kalra et al., 2012; Kim et al., 2013).

**FIGURE 2:**
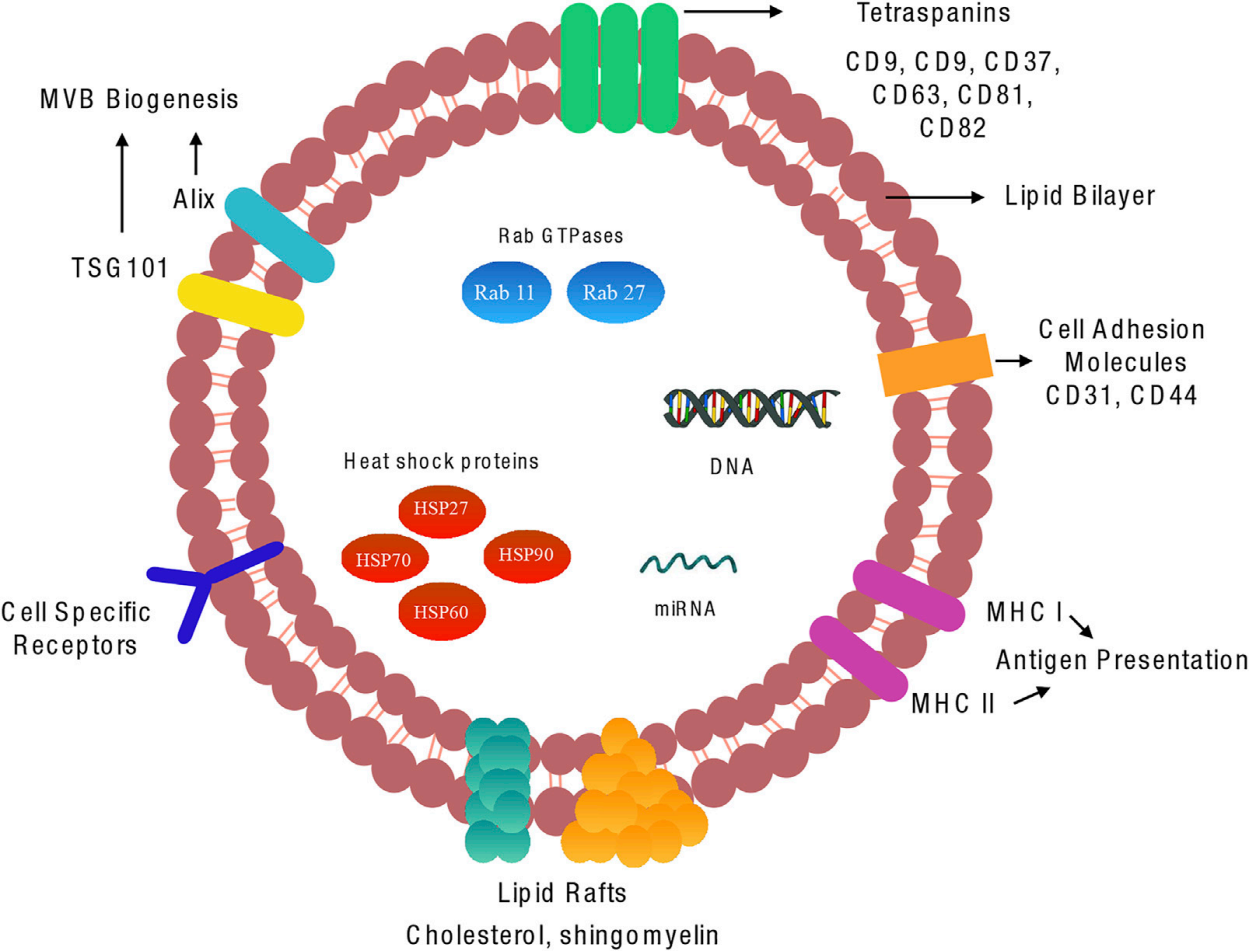
Molecular components of vascular exosome: Exosomes are composed of a plasma membrane-derived phospholipid bilayer containing cytosol components from the cell of origin. The composition of the exosomes is dependent on the cell type of origin, the state of health of the source cell, and extracellular stimuli. There are various proteins, lipids, and miRNAs that are common to the majority of exosomes.

Although exosomes contain a comprehensive and heterozygous range of molecules, there are some aspects of exosome structure that are generally conserved across many populations. Scattered amongst their lipid bilayer is a cohort of proteins with various functions. Amongst these proteins are the tetraspanin family, typically CD9, CD37, CD63, CD81 and CD82 that are involved in multiple functions such as cell penetration, invasion, and fusion (Escola et al., 1998) (Figure 2).

Although CD9 was the first of the family of tetraspanins discovered in dendritic cells, several studies have reported an abundance of CD63 and CD81 expressed across a broad range of exosomes; these proteins are therefore considered to be robust markers for exosome detection. Also present within exosomes are small heat shock proteins (HSP27, HSP60, HSP70 and HSP90) that control the cellular response and antigen presentation during cellular stress, in addition to assisting protein folding and trafficking (Srivastava, 2002). Multivesicular body formation proteins involved in exosomes formation and release, Alix, TSG101, Rab GTPases such as RAB11B, RAB27A and ARF6, are also abundant within exosomes. Interestingly, endoplasmic reticulum, Golgi apparatus and nuclear proteins have not been detected in exosomal fractions; however, numerous studies have shown the presence of key transcription factors involved in Notch, Wnt and Hedgehog signalling in exosomes (Kalra et al., 2012; McGough and Vincent, 2016). While these proteins are commonly encapsulated in exosomes, it has been suggested that they are not uniformly distributed in all subpopulations, therefore highlighting that even the same cell can release structurally homogeneous vesicles containing heterogeneous contents.

### Exosome Secretion and Release

Exosomes are released during physiological and pathological conditions by many cell types. There are two main pathways by which exosomes are released and secreted from their host MVB: the constitutive release pathway, associated with general physiological secretion of exosomes (Kalluri and LeBleu, 2020); and/or the inducible release pathway, initiated by stimuli such as heat shock, hypoxia, DNA damage, increased intracellular calcium release and lipopolysaccharide. The initiation of exosome release begins with the transport of MVBs to the plasma membrane, with this process depending on their interaction with actin and the microtubule cytoskeleton (Sinha et al., 2016).

There are many mediators involved during membrane trafficking of ILVs (including vesicle budding, membrane fusion, and transport along actin and tubulin) (Giannitsis et al., 2019). The most important of which are Rab guanosine triphosphatases (Rab GTPase), the largest family of small GTPases composed of approximately 70 distinct proteins (Stenmark, 2009). Among these are Rab11 and Rab35, that control trafficking membrane cargo to and from recycling endosomes on the way to the plasma membrane. Rab27A, promotes docking of MVBs and fusion to the plasma membrane while Rab27B facilitates vesicle transfer from the Golgi to MVBs (Ostrowski et al., 2010). In 2002 Savina et al. (2002) were the first to show the role of the Rab GTPase, Rab11 in exosomes secretion in human leukaemic K562 cells using ectopic expression of a dominant-negative Rab11 mutant to inhibit exosome release. Similarly, Rab11 depletion in *drosophila* S2 cells attenuated exosome secretion (Koles et al., 2012). Furthermore, shRNA knockdown of Rab2b, Rab GTPases in HeLa cells revealed that depletion of Rab27a and Rab27b reduced the ability of MVBs to dock onto the plasma membrane (Ostrowski et al., 2010) while Rab35 controls MVB docking and membrane fusion in oligodendrocytes (Hsu et al., 2010). By contrast, there are several GTPase independent pathways involved in exosome release, including through hypoxia-inducible factor 1α (King et al., 2012), glycan synthase kinase 3 (Hooper et al., 2012) and activation of p53 (Lespagnol et al., 2008).

Once transported to the plasma membrane, MVBs undergo a fusion event followed by exosome secretion into the extracellular space. In order to allow these coordinated multilevel changes in cytoskeletal plasma, membrane interactions have to be overcome. The first such interaction involves energy reduction of the membrane through protein-protein and protein-lipid interactions and enzymatic activation of membrane fusion machinery. Soluble *N*-ethylmaleimide-sensitive factor attachment protein receptors (SNAREs), tethering factors, Rabs, and other Ras GTPases facilitate MVBs fusion with the plasma membrane. To date, the exact molecular requirements for MVB membrane fusion are not well established; however, many studies have reported the crucial role of SNARE proteins and synaptotagmin family members (Jahn and Scheller, 2006). The primary function of SNARE proteins and their associated complexes is to facilitate vesicle fusion to the plasma membrane while providing specificity for membrane trafficking *via* the formation of a SNARE complex. Members of this family of proteins are classified into two categories, R or Q SNAREs. The formation of the SNARE complex involved in membrane fusion includes one R-SNARE, vesicle-associated membrane protein 7 (VAMP7) and three Q-SNAREs forming four coiled-coil helices (Pfeffer, 2007). RSNARE proteins VAMP7 and YKT6 have been reported as a necessity during exosome fusion of leukemic K562 cell lines and HEK293 cells, respectively (Fader et al., 2009).

During membrane fusion, vesicle SNAREs localized on MVBs interact directly with the target SNAREs to form a SNARE complex. Once this complex is formed, the MVB successfully fuses with the plasma membrane. Importantly, it has been shown that exosome secretion can be initiated independent of Rab GTPases through increases in intracellular calcium levels (Ca^2+^). Increased levels of intracellular calcium following monensin or calcium ionophore A23187 increases exosome secretion in human erythroleukemia K562 cells (Savina et al., 2003). Similarly, Fauré et al. reported that following potassium-induced depolarisation of critical neurons, subsequent influx of intracellular calcium led to an increase in exosome secretion. Comparable findings have since emerged for oligodendrocytes and breast carcinoma cell lines (Messenger et al., 2018). It is also noteworthy that some proteins, such as the synaptotagmin family, function as calcium sensors controlling exosomes secretion whereby knockdown of synaptotagmin-7 reduces the rate of exosome secretion (Hoshino et al., 2013). Regardless of the mechanism by which they are released from MVBs, exosomes remain close to their host cell interacting with neighbouring cells or are transported through bodily fluids to distant target cells.

### Exosome Uptake and Function

**FIGURE 3:**
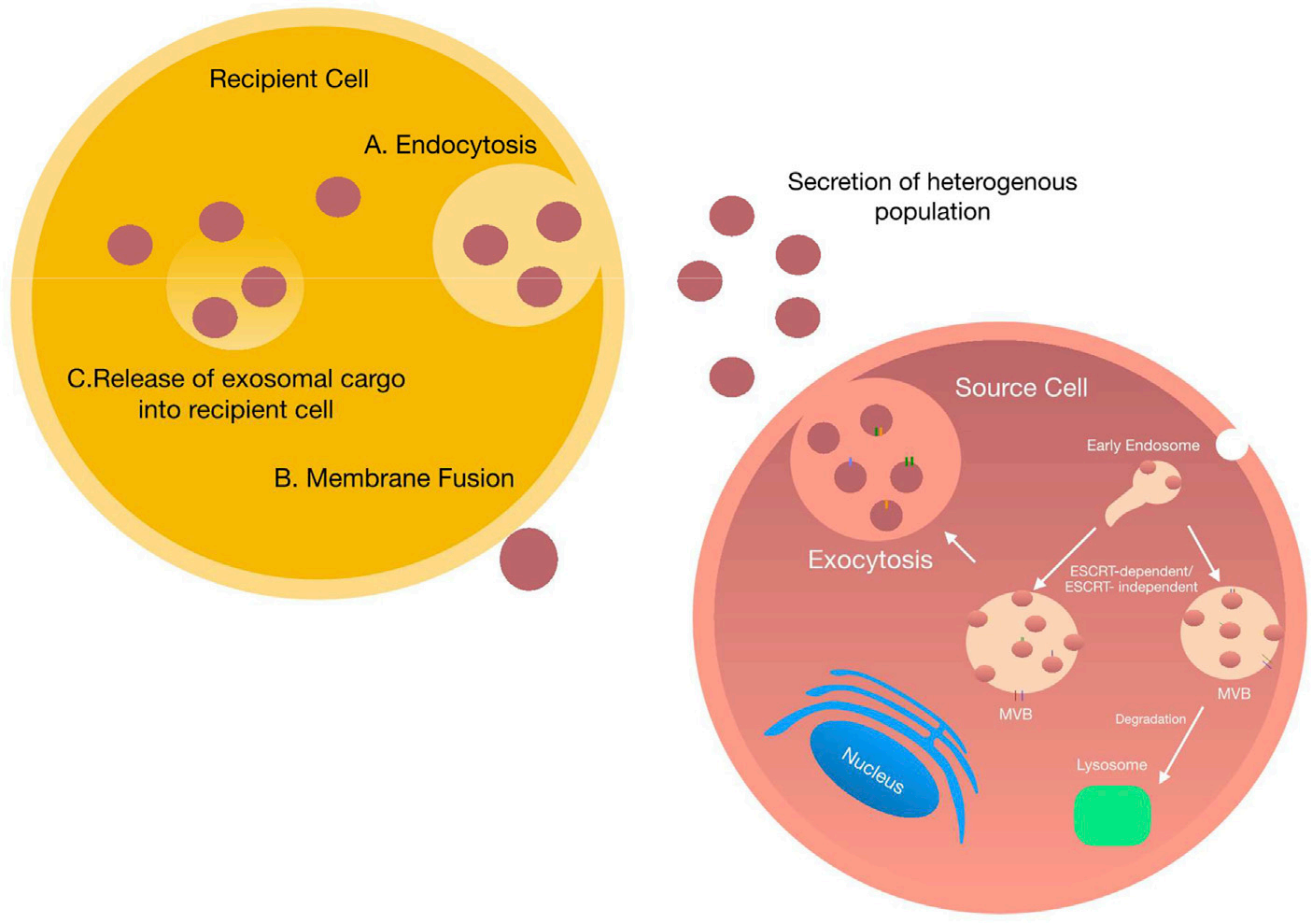
Exosome uptake by neighbouring cells: Schematic representation of exosome uptake by neighbouring cells. Once secreted into the extracellular space, exosomes can be taken up by neighbouring cells *via* two main processes i) exosomes are engulphed into the target cell *via* endocytosis. Once endocytosed exosomes fuse with the endocytic compartment delivering various cargo ii) exosomes dock onto the plasma membrane of the target cell and directly deliver their cargo into the cytoplasm of the target cell.

On release, exosomes arrive at their target cell and present their contents to initiate functional responses, and/or promote phenotypic changes affecting the physiological or pathological status of the cell. Current research suggests that successful delivery of exosomal cargo can occur by two main mechanisms—*1*) endocytosis or *2*) direct fusion with the target cell (Figure 3). Exosomal endocytosis is a multi-step process that begins with exosome docking onto the plasma membrane of the recipient cell *via* receptor-ligand binding. Although the precise mechanisms for specific targeting of recipient cells are not clearly defined, several mediators are known, including specific lipids, lectins, tetraspanins, various extracellular matrix components and heparan sulfate proteoglycans. For example, uptake of tumour and non-tumour derived exosomes is substantially reduced in following the block of heparan sulfate proteoglycans (Atai et al., 2013; Svensson et al., 2013). Similarly, presentation of integrins on exosomes interact with cell adhesion molecules at the surface of recipient cells facilitating stable docking and attachment.

Irrespective of the mechanism by which exosomes dock to the recipient cell, once bound they either remain attached to the plasma membrane or become internalised (Denzer et al., 2000; Buschow et al., 2009). The method by which the exosomes become internalised differs depending on the cell type. Multiple mechanisms of endocytosis have been shown; for example, phagocytosis and clatherin-mediated endocytosis in neurons, macro-pinocytosis by microglia, caveolin-mediated endocytosis in epithelial cells and lastly lipid-raft endocytosis in tumour cells (Barrès et al., 2010; Feng et al., 2010; Fitzner et al., 2011; Nanbo et al., 2013).

Alternatively, exosomal uptake can occur *via* a direct membrane fusion event; however, this requires insertion of fusogenic proteins into the plasma membrane, followed by lipid reorganisation, protein reconstruction and membrane dimpling. Following interaction and uptake by recipient cells, exosomes may fuse with internal cytoplasmic endosomes resulting in the transfer of bioactive materials or possible degradation. However, it is currently unclear exactly how the cargo transfer takes place (Abels and Breakefield, 2016). Once internalised by the recipient cell, exosomes can initiate a diverse array of biological functions in both physiological and pathological conditions. Intercellular communication, immune modulation, coagulation and thrombosis and protection against cellular stress and death all represent mechanisms initiated in physiological conditions (Peters et al., 1989; Zitvogel et al., 1998; Valadi et al., 2007; Verma et al., 2015).

## Endothelial Exosomes

**FIGURE 4:**
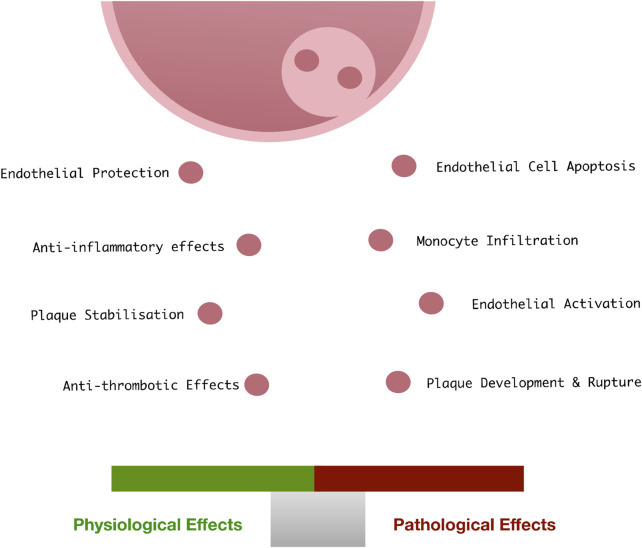
Endothelial exosomes in vascular health and disease: Under physiological conditions, endothelial cells release exosomes at low concentrations which facilitate the protection and vascular homeostasis. When exposed to pathological vascular stimuli, the content of these exosomes changes, resulting in a loss of vascular homeostasis, apoptosis, monocyte infiltration, and subsequent facilitation of the development of CVD.

Like most cells in the body, vascular ECs retain the ability to release exosomes into the extracellular space in response to cellular activation or apoptosis. Vascular endothelial-derived exosomes play a role in physiological processes maintaining vascular homeostasis (Ridger et al., 2017), vascular development (Sheldon et al., 2010), endothelial regeneration and vascular cell protection, as well as the pathological progression of vascular disease (Bellin et al., 2019) (Figure 4). They are characterised by expression of various EC specific surface markers (CD31, CD55, CD144 and von Willebrand factor).

### Endothelial Exosomes in Cardiovascular Disease

It is widely accepted that when exposed to cellular stress or oxidative damage, ECs release growth factors and cytokines which render change amongst neighbouring vascular cells (Haller et al., 2020). Invariably, ED leading to EC apoptosis drives maladaptive responses by favouring the release of atherogenic factors leading to perturbation of vascular homeostasis and vessel remodelling (Choy et al., 2001). Whether these bioactive materials are packaged into an exosome and secreted to impact other cells remains under active investigation. Nevertheless, it has recently been reported that circulating EC-EVs may differentiate STEMI from NSTEMI patients (Galeano-Otero et al., 2020; Haller et al., 2020). As guardians of vascular homeostasis, vascular ECs secrete low concentrations of exosomes into the bloodstream under physiological conditions. However, upon induction of ED, the concentration and content of EC-derived exosomes may change significantly whereby exosome mediated cell-cell communication is thought to drive vascular pathology (Migneault et al., 2020). Their biological effects are extremely complex and depend on the dysfunctional state of the releasing EC, their protein and nucleic acid content, and the diverse nature of the recipient cells (SMCs, ECs, macrophages/monocytes, stem cells).

Many studies have shown an increase in exosome content during the onset and progression of diabetes mellitus, atherosclerosis and MI (Mallat et al., 2000; Bernal-Mizrachi et al., 2003). The release of exosomes from dysfunctional ECs mediate important repercussions on overall vascular function and pathology [cellular senescence, apoptosis and vascular calcification (VC)] due to their inherent capacity to easily access the bloodstream, resist degradation and significantly impact neighbouring cells within the vessel wall. Despite multiple studies demonstrating an association between endothelial release of circulating EVs and CVD, the understanding and functional implications of exosome secretion and cargo content are less well known. Indeed, the uptake of EC-derived exosomes from dysfunctional ECs containing various transcription factor proteins, lipids and miRNAs initiates a functional response in neighbouring cells. Quantitative proteomic analyses reveal that exosomes derived from ECs under different culture conditions have unique cargo (de Jong et al., 2012). *In vitro,* EC-EVs can be induced by TNF⍺, IL-1β, thrombin, lipopolysaccharide (LPS), C-reactive protein, and plasminogen activator-1 (Hromada et al., 2017). In addition, laminar shear stress, hyperglycaemia, and hypoxia are strong inducers of EC-EV generation (Hromada et al., 2017). Using *in vivo* models of ischemia–reperfusion injury, the release of exosomes from dysfunctional ECs within the circulation has also been demonstrated (Dieudé et al., 2015). These EVs, termed ApoExo’s are released by human and murine ECs *in vitro* and characterized by the expression of an active 20 S proteasome core complex regulating their immunogenic activity to induce a pro-inflammatory response in naïve and transplanted mice (Dieudé et al., 2015; Migneault et al., 2020). Exosomes derived from dysfunctional ECs may also promote anti-apoptotic events in neighbouring cells through NF-κB activation thereby mediating a pro-survival, pro-migratory and anti-angiogenic phenotype in ECs consistent with changes to endothelial phenotype of potential importance in vascular response to injury (Migneault et al., 2020). In addition, KLF4-induced secretion of exosomes from human umbilical vein endothelial cells (HUVECs) harbours miR-143 and miR-145 enriched cargo which, upon uptake by vascular SMCs, regulate their phenotype through changes in vascular de-differentiation genes such as ELK1, KLF4, CAMK2d, and SHH2. Furthermore, exosomes derived from ECs expressing KLF4 also induced the formation of atherosclerotic lesions (Hergenreider et al., 2012). Exosome secretion is also particularly important in senescent ECs. Exposure of young ECs to exosomes from senescent ECs decreased the expression of inter-endothelial adherens junction proteins, VE-cadherin and beta-catenin (Takasugi, 2018) leading to altered endothelial junctional communication including suppressed cell growth, proliferation and migration of ECs that disrupt the endothelial barrier integrity (Wong et al., 2019).

A similar cross-talk mechanism exists between vascular ECs and circulating monocyte/macrophages, promoting the formation of atherosclerotic plaque. Exosomes secreted by LPS/interferon-alpha activated monocytes are endocytosed by human endothelial cells resulting in the expression of ICAM-1 and pro-inflammatory cytokines. This process results in ED *via* the Toll-like 4 (TLR4) and nuclear factor kappa-light-chain-enhancer of activated B cells (NF-κB) pathways contributing to the development of heart disease and potentially other chronic diseases (Tang et al., 2016). This exosome-dependent monocyte to endothelial crosstalk is also operational between monocytes and ECs and visa-versa in response to high glucose through modulation of the expression of surface proteins such as intercellular adhesion molecule 1 (ICAM-1) (Sáez et al., 2019). These findings support previous data showing that circulating EVs from newly diagnosed diabetes type 2 patients are enriched with proteins responsible for cell activation, i.e., ICAM-1 (Mallia et al., 2020). It has also been shown that transcription factors as components of the Notch and Wnt signalling pathways, such as delta-like 4 (Dll-4), are transported *via* exosomes to manage angiogenesis, a fundamental process in CVD (Gross et al., 2012; McGough and Vincent, 2016). Dll-4 is present in exosomes secreted by vascular ECs and is taken-up by neighbouring ECs to promote angiogenesis though inhibition of the Notch signalling pathway (Ushio-Fukai and Urao, 2009).

Numerous studies have shown that exosomes share many characteristics of matrix vesicles (MVs), which are unique extracellular membrane-bound microparticles that serve as initial sites for mineral formation (Qin et al., 2021). Exosomes are important transporters of molecules that drive vascular calcification (VC). VC is the abnormal deposition of calcium, phosphorus, and other minerals within the vessel wall and is closely associated with lesion formation and increased mortality due to pathological cardiovascular events. Both exosomes and MVs are enriched in calcified vascular walls and may promote the occurrence of VC through mineral deposition, and the reduced expression of fetuin-A (a known calcification inhibitor) in secreted exosomes (Qin et al., 2021). The current understanding on the origin and functions of osteoblast-like and osteoclast-like cells in the development and progression of calcified vascular lesions has been recently reviewed (Jiang et al., 2021). While smooth muscle trans-differentiation leading to VC in atherosclerosis is widely cited, recent focus on circulating and resident progenitor cells in the vasculature and their role in atherogenesis and VC has emerged (Leszczynska and Mary Murphy, 2018). When exposed to inflammatory stimuli, pericytes in particular, may turn into mesenchymal stromal progenitors and develop into osteoblasts and chondrocytes and produce large nodules containing type I collagen, osteopontin, matrix Gla protein (MGP), and osteocalcin to promote arterial calcification (Canfield et al., 2000; Leszczynska and Mary Murphy, 2018). The molecular mechanisms and balance between osteoblast-like and osteoclast-like cells in driving vascular calcification appears decisive (Zhang et al., 2018). Changes of miRNAs in exosomes can regulate osteogenic differentiation by promoting the expression of Runx2 and activating related signalling pathways, for example, the Wnt/β-catenin pathways (Leszczynska and Mary Murphy, 2018). Studies have shown that miRNAs with increased expression in the VC process can promote the osteogenic transformation of pericytes and SMCs by targeting anti-calcification proteins or contractility markers, while miRNAs with decreased expression can inhibit the osteogenic transformation by targeting osteogenic transcription factors (Zhang et al., 2018). Indeed, exosomes released from glucose-treated human ECs contain Notch3, an important receptor in cell differentiation, and are able to promote vascular calcification through the mammalian target of rapamycin signalling pathway (Lin et al., 2019).

Other important mechanisms driving exosomal control of VC include regulation on cell autophagy (Dai et al., 2013) and oxidative stress (Chang et al., 2008). The autophagy pathway is a key regulator of cellular metabolism and EC homeostasis, and plays a critical role in maintaining normal vascular cell function (Phadwal et al., 2020). Emerging evidence suggests that autophagy directly protects against VC where endosomes, dysfunctional mitochondria, autophagic vesicles and Ca2+ and phosphate (Pi) enriched matrix laden EVs may work collectively to underpin the pathogenesis of VC. Excess ROS-induced stress has emerged as a critical mediator promoting VC through several mechanisms, including phosphate balance, phenotypic modulation of vSMCs, inflammation, DNA damage, and extracellular matrix remodelling (Hu et al., 2021).

In summary, a large body of research provides substantial evidence in favour of the active involvement of endothelium-derived exosomes in the development and progression of associated vascular diseases. Moreover, as endothelial exosomes efficiently deliver their cargo into recipient vascular cells to change function, they represent an important potential biomarker for disease detection and a therapeutic agent to effectively treat disease.

### Exosomes and Their Diagnostic Potential

As exosomes are remarkably stable in biofluids, such as plasma and urine, and can be isolated for clinical evaluation even in the early stages of the disease, exosome-based biomarkers have quickly become adopted in the clinical arena with the first exosome RNA-based prostate cancer test introduced for early prostate cancer detection (Yu et al., 2021). Currently, circulating biomarkers of CVDs, such as total cholesterol levels and LDL, and MI prognostic biomarkers, including high-sensitivity C-reactive protein, high-sensitivity cardiac troponin and creatine kinase MB evaluate the risk of the occurrence and progression of the disease but fail to precisely predict whether the process starts or develops (Howard et al., 2000).

In the past decade, several studies have shown an association between elevated numbers of EVs, including exosomes, and prediction and development of CVD. Circulating EVs have been described during the development of atherosclerotic plaques (Aikawa, 2016; Kapustin and Shanahan, 2016), peripheral arterial disease (Badimon et al., 2016) and are associated with the Framingham risk assessment score (FRS) to determine a patient’s 10-year risk of developing CVD. Currently, classical biomarkers used to diagnose acute pathologies associated with CVDs, such as myocardial infarction, are cardiac troponin I (cTnI) and isoenzyme creatine kinase MB. These biomarkers generally peak 12 h from the onset of an acute myocardial event, and their circulating levels are directly proportional to the scale of the infarct event (Adams et al., 1994). MiRNAs are the most widely studied element of exosome cargo. The importance of upregulation of two cardiac-specific miRNAs, miR-1 and miR-133a has been highlighted is studies using serum from patients diagnosed with an acute coronary event (Kuwabara et al., 2011). The levels of miR-1, mi-R133a, as well as mi-R208b and miR-499 were significantly increased in these patients compared to healthy volunteers. However, when compared to cardiac troponin T (cTnT), the four miRNAs were inferior for the diagnosis of AMI. By contrast, circulating miR-208a, which was undetectable in healthy patients, was elevated in 100% of patients presenting with MI and was detected within 4 h from the onset of chest pain, reaching its peak earlier than that of cardiac troponin (cTn) (Wang et al., 2010). Despite the identification of many other miRNAs as possible diagnostic biomarkers of atherosclerosis has-mi-R-192 and has-miR034a have shown the greatest promise as an early prognostic marker of disease. These miRs are upregulated in serum samples from AMI and ACS patients that experienced acute cardiovascular events within a year of their detection and support a role for exosome encapsulated miRs as biomarkers for both CVD and the prediction of adverse health outcomes.

Liquid biopsy-based exosomal profiling offers a promising platform to assist clinical diagnosis and prediction more accurately. In particular, exosomal miRNAs possess promising protective functions in CVD while circulating exosomes have shown great potential for diagnosis and risk assessment in CVD. In particular, substantial progress has been made in identifying the therapeutic role of circulating EC-derived EV miRNAs, particularly miR-92a-3p, in regulating the phenotype of ECs and SMCs under atherosclerotic conditions (Liu et al., 2019). Furthermore, miR-939-5p is downregulated in serum-based exosomes from patients with MI (Li et al., 2018).

Apart from miRNAs, exosomal proteins may also play a significant role in CVD diagnostics. P-selectin-expressing microparticles, CD3/CD45, SMA-α circulating exosome levels, and exosomal cystatin C, Serpin F2, CD14 levels are all significantly correlated with a high risk for incident CVD and mortality (Kanhai et al., 2013). In all, 252 upregulated EV proteins including apolipoprotein C-III, apolipoprotein D, platelet glycoprotein Ib alpha chain, complement C1q subcomponent subunit A, and complement C5 have been identified after MI using liquid chromatography coupled to tandem mass spectrometry and present an important opportunity for an EV diagnostic panel for the early diagnosis of MI (Cheow et al., 2016).

Similar studies have been performed in heart failure patients where miR-22, miR-320a, miR-423-5p, and miR-92b levels in both serum and serum exosomes present as potential specific biomarkers for the diagnosis and prognosis of systolic HF (Goren et al., 2012). Several serum-based exosomes containing p53-responsive miRNAs, such as miR-34a, miR-192, and miR-194 are also upregulated in HF patients within 1 year of acute MI onset (Matsumoto et al., 2013). Furthermore, an increased ratio of endothelial apoptotic microparticles (CD31^+^/Annexin V^+^) to mononuclear progenitor cells is elated to adverse clinical outcome in patients with acutely decompensated chronic HF (Berezin et al., 2015).

## Cardiovascular Diagnostics

The measurement of biomarkers is a familiar feature of cardiovascular assessments that often dictates the provision of additional diagnostic procedures and subsequent therapy. Their evaluation is integral to the rule-in/rule-out of AMI and they are frequently utilised to generate a cardiovascular risk score, which can increase the efficiency of the screening procedure (Papendick et al., 2017; Giannitsis et al., 2019), and consequently promote cost savings (Riley et al., 2017). Biomarkers are also finding utility in companion diagnostics and precision medicine, aligning with specific therapies and combining with molecular data to direct clinical decision-making (Currie and Delles, 2018; Hampel et al., 2019; Welcher, 2019). Their expanding applicability has accelerated research to further understand their relationship with cardiovascular injury and has promoted the pursuit for novel biomarkers of CVDs. Crucially, incorporating exosome measurement within diagnostic assessments in clinical settings will depend upon the development of fitting devices to enable rapid and/or high-throughput analysis, potentially demanding adapted processing procedures.

Currently, EVs are enriched and recovered using techniques such as centrifugation (ultracentrifugation, differential and gradient centrifugation), polymer-based precipitation, immunoaffinity, chromatography and filtration and are often chemically lysed after isolation (Doyle and Wang, 2019). Moreover, subsequent analysis can be conducted to recognise the released biomarker and accurately measure its presence—the exact manner of which depends upon the specific analyte. As a result, the entire analytical process can last several hours. This illustrates the resource intensive nature of measuring exosomes and their contents, suggesting this general approach is inherently unsuited to clinically urgent applications. Furthermore, although some exosomes may indicate pathological changes attributed to a CVD, to justify incorporation among current diagnostic techniques, their analysis should support the recognition of CVDs and ultimately improve patient management.

### Point-of-Care Testing

Hospitals and clinics rely heavily on central laboratories to analyse patient samples and deliver accurate and timely results. This common operational model places the vast majority of blood/sample analysis responsibility on the central laboratory, irrespective of the relative exigency of the test. However, the layout of this model, in which sample transport and handling contribute to delays, is unfavourable for streamlining the triage process and ensuring critical needs are fulfilled. By contrast, point-of-care-testing (POCT) requires conducting a diagnostic test in a patient’s proximity and incorporates the use of a compact device to facilitate rapid biomarker measurement. Unlike central laboratories in which a variety of sophisticated instruments and measurement equipment are routinely utilised, a point-of-care (POC) platform is generally robust, with the capacity to perform measurements in a range of test environments and without the need for highly specialised training.

This diagnostic approach is ideally suited to cardiovascular assessments in which immediate action is a priority. For instance, minimising the system delay—which is the time period between emergency department presentation and insertion of a catheter—correlates with reduced infarct size and improved left ventricular function following myocardial injury (Lonborg et al., 2012). A prominent characteristic of POC testing (POCT) is the ability to accommodate rapid analysis, ideally generating a response within 20 min of sample application. This rapid response has been shown to contribute towards reduced turnaround times (TATs), with one study demonstrating that the implementation of POCT for cTnI, the primary biomarker of AMI, reduced the length of stay by over 25% in emergency departments, thus shortening the system delay (Singer et al., 2005). This is one of many facets of POCT that rely on key characteristics for implementation . However, several criteria are identified including *1*) “laboratory testing is performed in the direct proximity of the patient,” *2*) “results available quickly” and *3*) “results lead to a rapid diagnosis or consequences for treatment.” Evidently, they all relate to improving patient management and the efficiency of the process. As a result, this format of diagnostic testing is ideally placed to positively impact cardiovascular assessments due to the critical need to triage patients without delay.

**FIGURE 5:**
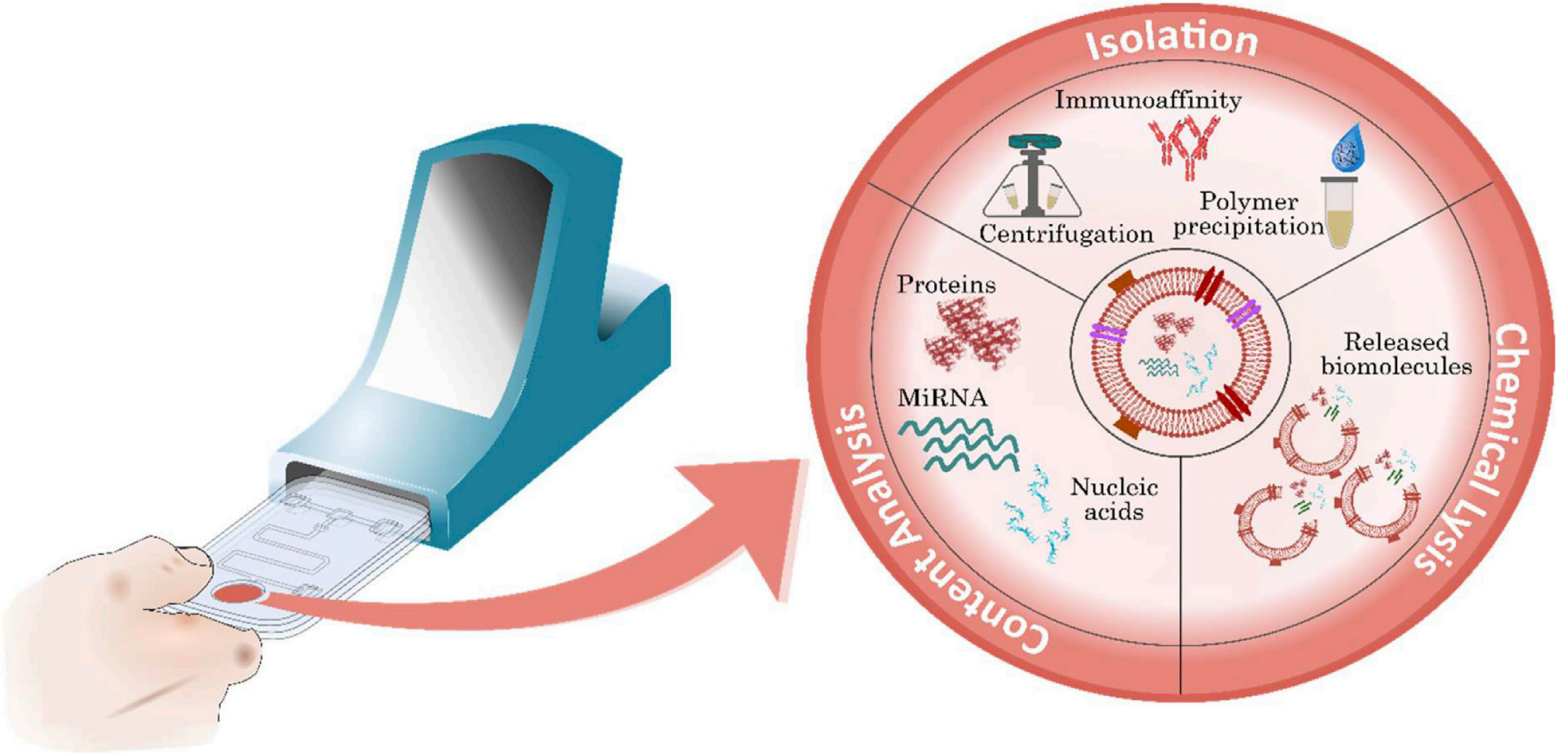
Exosome-derived biomarkers for the diagnosis of CVD. Exosomes contain key potential biomarkers including various surface proteins, miRNAs and nucleic acids. Following isolation, exosomes can be measured, or further processed using exosome lysis before biomarker recognition to analyse contents.

The development of compact and user-friendly platforms that can accommodate exosome analysis, whilst adhering to quality assurance and compliance with accreditation standards, in addition to the aforementioned recommendations, requires a combination of meticulous design and careful consideration of both the isolation and measurement processes (Figure 5). Moreover, the simplistic operation of these devices is made possible by the miniaturisation and automation of sequential tasks often associated with laboratory assays. Importantly, some processes are naturally more suited to POCT and are somewhat unrestricted by technological limitations. These are key factors to contemplate when assessing the feasibility of exosome measurement for POCT as current isolation processes and measurement techniques may be inherently unsuited for POCT.

## Exosome Detection for Cardiovascular Disease Diagnostics

The isolation of pure exosomes is a critical step in developing exosomal profiling for clinical diagnostic application in CVD. There is a lack of consensus regarding the most appropriate approach for exosome isolation and characterization, both important issues for their effective clinical translation. A major challenge is the identification of a standard technique to isolate intact exosomes from bodily fluids with high reproducibility and purity. The various isolation platforms have been recently reviewed and include: *1*) Ultracentrifugation, *2*) Size-Based Isolation Methods *3*) Immuno-Affinity Purification *4*) Polymer-Based Precipitation and *5*) Microfluidics-Based Isolation Techniques (Zarà et al., 2020) (Table 1). Conventional exosome isolation processes such as centrifugation (most widely implemented) (Gardiner et al., 2016) and some polymer precipitation methods can take over 12 h to perform and are often reported to produce low recovery rates and result in an impure extract (Patel et al., 2019). Exosome purity and yield can be affected by several factors including rotor type, *g*-force and centrifugation times thus compromising centrifugation as a recognised standard for clinical exosome isolation (Cvjetkovic et al., 2014). Moreover, the purity of the exosome product is entirely dependent upon the isolation technique with a combination of ultrafiltration and chromatography producing much higher purity exosome yields than centrifugation (Shu et al., 2020), although this may only exemplify the challenges facing standardisation in practical applications.

**TABLE 1:** Performance characteristics of highly specific exosome isolation techniques.

Isolation technique	Property of separation	Specimen type	Sample volume capacity (µl)	Isolation time (min)	Particle size (nm)	Recovery rate
Dielectrophoresis (Shi et al., 2019)	Dielectric properties shape and size of particles in fluid	Plasma (filtered), serum and saliva (centrifuged and pre-concentrated)	200	20	50–150	NA
Dielectrophoresis (Ibsen et al., 2017)	Dielectric properties shape and size of particles in fluid	Plasma	30–50	30	50–150	NA
Microfluidic gel electrophoresis and ion-selective separation (Marczak et al., 2018)	Surface properties and particle size	Serum	25–50	10–20	130–260	60–80%
Ion concentration polarization with 3D-printed microtrap (Cheung et al., 2018)	Electrophoretic mobility of EVs, particle size/immunoaffinity and hydrophobic interactions (aldehyde)	EVs in PBS	30	30	Average size ≈50–75	Concentrate 100-fold
Acoustic trapping (Ku et al., 2018)	Particle size, density and compressibility of particles and fluid	Plasma and urine	300	30	154.2 (mean)	2.4*10^8 particles/ml
Immunomagnetic (Zhao et al., 2016)	Immunoaffinity	Plasma	10—10,000	20	79.7% < 150	72%
Immunocapture (Zhang et al., 2019b)	Immunoaffinity	Plasma	2–125	40 for 20 µl sample (0.5 µl/min)	40–160	80–85%
Fe_3_O_4_@TiO_2_ particle enrichment (Pang et al., 2020)	Affinity of phosphate head to TiO_2_ and particle size	Serum (filtered)	4	5	30–200	NA
TiO_2_ particle enrichment (Gao et al., 2019)	Affinity of phosphate head to TiO_2_	Serum (filtered)	1–100	5	65–235	93.4
Tim4- phosphatidylserine affinity (Nakai et al., 2016)	Affinity of Tim4 protein towards phosphatidylserine	Cell culture supernatant (filtered)	50—4,000	Overnight	219 (mean)	15—20%
Nanostructure- functionalized lipid nanoprobe (Wan et al., 2019)	Affinity and particle size	Plasma (filtered)	1,000—2,000	100–200 (10 µl/min)	50–200	28.8%

Many of the techniques selected display favourable features for adaption within compact microfluidic-based cartridges. Bodily fluid sample often filtered to remove cellular debris and large extracellular vesicles. Fe_3_O_4_, Iron oxide, TiO_2_, Titanium dioxide.

### Electrokinetic Separation

There are alternative isolation methods which not only offer improved performances but are more suited to POCT for CVD diagnostics. Dielectrophoresis (DEP), for instance, is a technique that enables controlled manipulation of particles in a fluid and can separate particles based on their dielectric properties, shape, size and the properties of the surrounding fluid using a non-uniform electrical field to induce a dipole moment in the targeted particles (Pethig, 2017). Moreover, it is ideally suited to separate EVs from bodily fluid samples and is widely utilised in a variety of microfluidic devices (Zhang et al., 2019a) and rapidly isolates exosomes from conditioned cell culture media, plasma, serum and urine in 20 min (Shi et al., 2019). This approach can be improved using insulator-based DEP to squeeze the electric field and avoids common electrode fouling and electrolysis problems associated with electrode-based DEP (Crowther and Hayes, 2017). Another DEP-based isolation device contains a microelectrode array to isolate exosomes from plasma samples and concentrate them at the microelectrode edges (Ibsen et al., 2017). Importantly, the microelectrode array consisted of a hydrogel coating to minimize fouling and reduce bubble formation, thus preserving the integrity of the electrode surfaces. A slightly different approach, which also utilises an electrical field for particle manipulation, is ion concentration polarization (ICP). Here, the electric field effectively drives the transportation of particles to the interface of an ion-exchange membrane at a rate dependent on the particle’s electrophoretic mobility in a particular fluid (Fu et al., 2018). However, as this technique alone does not strictly discriminate between EVs and other biological fluid particles, ICP is often implemented in conjunction with complementary separation techniques (Marczak et al., 2018). This technique concentrates exosomes from serum within 10 min to a factor of 15 and could produce yields of approximately 70% in 20 min. Although, one potential limitation of this device is that the entire buffer solution must be exchanged frequently during operation. Another approach is to incorporate multiple separation mechanisms to enhance the recovery efficiency of isolation techniques. The development of an ICP-based microfluidic device that consists of 3D printed microtraps to assist exosome capture (Cheung et al., 2018) results in a 100-fold increase in the exosome concentration within the microchannel in 30 min.

### Affinity-Based Isolation

Employing antibodies to target specific surface proteins is one of the more common approaches frequently reported for exosome isolation. Several studies detail the development of microfluidic-based EV enrichment using antibodies and demonstrate performance enhancement techniques such as; magnetic nanoparticle (Zhao et al., 2016) or magnetic nanowire conjugation of antibodies for improved particle manipulation (Lim et al., 2019), optimised microfluidic design to maximise sample mixing (Zhang et al., 2016; Dorayappan et al., 2019) and the use of microstructures (Zhang et al., 2019b) and nanomaterials (Fang et al., 2017) to optimise the antibody loading capacity by increasing the overall surface area. However, EVs display other distinctive properties which can support alternative affinity-based interactions. These include the attraction between phospholipids and TiO_2_ to facilitate EV isolation. Fe_3_O_4_@TiO_2_ core-shell nanoparticles were synthesised, in which the outer TiO_2_ shell supported binding of serum exosomes, whereas the Fe_3_O_4_ core enabled particle manipulation by magnetic separation from the sample and hence, exhibits some favourable characteristics for POCT (Li et al., 2017; Pang et al., 2020). Synthesis of TiO_2_ -containing microspheres to facilitate TiO_2_-based isolation of exosomes from buffer solutions and serum within 5 min has also been reported (Gao et al., 2019). However, a potential limitation of TiO_2_ exosome isolation is the lack of specificity which may produce low purity extracts as serum proteins have also been shown to interact with TiO_2_ nanomaterials (Zaqout et al., 2011; Kulkarni et al., 2015). Hence, the purity of the exosome extract might be compromised, thus possibly affecting downstream analysis. Nonetheless, both TiO_2_-based isolation techniques were shown to achieve significantly improved performances in comparison to ultracentrifugation, with the second group performing proteomic analysis to demonstrate increased purity of the extract compared to samples processed by ultracentrifugation. Similar affinity-based approaches have also been reported, with one such technique immobilizing T-cell immunoglobulin domain mucin domain-containing protein 4 (Tim4) on the surface of magnetic beads to bind to phosphatidylserine (Nakai et al., 2016; Lea et al., 2017), a phospholipid typically found internally within the phospholipid membrane of vesicles. This technique has the distinct advantage of simple exosome release following enrichment due to the dependency of phosphatidylserine on the presence of Ca^2+^ (Tietjen et al., 2014). Although, the purity of the extracted exosomes acquired was higher (78.1%) than both ultracentrifugation (66%) and polymer precipitation (21.8%), one key limitation is that the exosome sample remains in the Tim4 protein overnight to initiate capture, an evident deficiency if to be considered for POCT.

The development of devices that incorporate lipid nanoprobes in conjunction with silica nanostructures to selectively isolate exosomes has also shown great promise (Wan et al., 2019). Reactive ion etching was employed to produce nanostructures in silica substrate correlating to EVs in the size range of approximately 120 nm, which also serves to increase the overall surface area available for lipid nanoprobe grafting. The lipid nanoprobe non-covalently interacts with the lipid membranes of the exosomes and thus together with the tailored nanostructures, offers an effective method for isolation. The microfluidic chip developed for the recovery of exosomes included a herringbone micromixer to stimulate increased interaction with the lipid nanoprobe functionalised nanostructures.

### Characteristics of Recent Isolation Techniques

The growing variety of highly specific EV isolation techniques provides an indication of the rate at which tailored approaches to EV isolation are emerging. Notably, many of these methods provide superior exosome yields compared to traditional isolation techniques and require less time to attain an enriched extract. A selection of EV isolation techniques and performance characteristics which are particularly relevant for POCT are summarised in Table 2. For instance, having the capacity to process small sample sizes is often a desired pre-requisite of POCT which permits the use of minimally invasive collection methods suitable for acquiring small sample volumes. On the contrary, processing larger amounts of sample may provide superior diagnostic value for exosome analysis. Nevertheless, performing sample analysis from small sample volumes with a short TATs are key characteristics of POC assays and are particularly relevant in acute care settings for CVDs. It is evident that the isolation techniques listed offer reduced processing time in comparison to the regularly employed standard approaches (Table 2). These techniques have been purposely developed to accommodate the separation of EVs from bodily fluids by targeting unique EV properties, with some implementing affinity–based approaches and others facilitating enrichment by particle size. However, the majority employ a combination of separation mechanisms which essentially augments the performance of these techniques, illustrating to an extent the existing inadequacies of traditional techniques due to their forced adaption for a process in which they are inherently constrained.

**TABLE 2:** Platforms for isolating exosomes directly from bodily fluid samples that are routinely collected in clinical settings.

Isolation technique	On-chip pre-treatment	Property of separation	Specimen type	Sample volume (µl)	Isolation time (min)	Particle size (nm)	Recovery rate
Tilted-angle standing surface acoustic wave (Reátegui et al., 2018)	None	Particle size, density and compressibility of particles and fluid	Whole blood	100	25	75–125	82.4%
On-disc AAO membrane filtration (Sunkara et al., 2019)	Centrifugal disc plasma separation	Particle size	Whole blood	30–600	36	100–350	76–88%
Standing surface acoustic waves (Wang et al., 2020)	None	Particle size, density and compressibility of particles and fluid	Saliva	NA	10–20	20–250	NA
DC electrophoresis-assisted filtration (Davies et al., 2012)	None	Size and electrophoretic mobility	Whole blood	240	120	NA	1.5%
Immunoaffinity (Chen et al., 2019)	Size exclusion membrane filtration	Affinity	Whole blood	20	500	50–200	45%
Immunoaffinity (Zhou et al., 2020)	Inertial separation	Affinity	Whole blood	75	78	50–200	NA
DEP (Lewis et al., 2018)	None	Dielectric properties, shape and size of particles in fluid	Whole blood	25	20	NA	NA

Automated on-chip pre-treatment involved removing components of whole blood to simplify recovery of exosomes. Isolation time includes any required pre-treatment. AAO, Anodic aluminium oxide; DC, Direct current.

The ability to recover a large quantity of exosomes from a sample is a fundamental aspect of an exosome isolation technique. This recovery rate (the ratio between extracted exosomes and total sample exosomes) is a key metric that can be assessed to validate the proficiency of an enrichment technique and thus support the direct comparison of different approaches. However, the recovery rate was not determined in several studies, thus restricting direct comparison with other approaches. The apparent lack of cohesion between reported performance characteristics of these exosome isolation techniques increases uncertainty regarding the most promising methods. Moreover, determining the purity of the enriched exosomes is quite important as it can assist in confirming that a biomarker has originated from EVs and not just been co-isolated with the exosomes (Webber and Clayton, 2013). The co-isolation of contaminating factors may inhibit accurate detection of the EVs and/or their contents, although, some consider the purity of the EV extract less important for diagnostic purposes, inferring that the yield is a primary concern (Quek et al., 2017; Konoshenko et al., 2018). Beyond diagnostic applications, exosome enrichment might be required for therapeutic purposes or biomarker discovery and therefore, a lack of cohesion between reported characteristics may only serve to delay the adoption of a recognised standard for exosome isolation.

### Exosomal Analysis for Point-of-Care-testing

A crucial aspect of adapting analytical techniques for POCT is the ability to refine and compartmentalise standard laboratory procedures to simplify the measurement process. A POC assay for instance, should measure biomarkers directly from samples without manual pre-treatment, thus eliminating the need for ancillary instrumentation and preventing inter-operator variability. Bodily fluid samples can be quite complex, however, often requiring pre-treatment to support accurate measurements. Whole blood, for example, comprises a multitude of biomolecules, cells and cellular debris which can be challenging to process. Therefore, plasma or serum are often the media of choice as red and white blood cells are removed, the main distinction between plasma and serum is that serum lacks the blood clotting agent fibrinogen. Crucially, before processing whole blood for serum extraction, the collected blood must be left to clot for approximately 30 min, whereas plasma separation can begin immediately, and can be complete within a few minutes. Various analytical approaches incorporate automated on-board plasma extraction prior to exosome isolation (*see* Table 2). These platforms permit exosome recovery from whole blood or saliva and can employ pre-treatment mechanisms to acquire plasma which minimises operator interaction and produces an enriched extract for subsequent biochemical analysis.

Centrifugal microfluidic lab-on-a-disc (LoaD) platforms are particularly suited to plasma extraction from whole blood with one group incorporating on-disc filtration to extract EVs from separated plasma (Sunkara et al., 2019). Whole blood samples were loaded onto the Exodisc-B LoaD platform and separated into individual constituents which enabled plasma to be collected for subsequent processing. This automated approach incorporated two filters that separated larger particles and smaller biomolecules from EVs within the plasma. The remaining EVs were collected in an elution chamber with a reported recovery efficiency that exceeded ultracentrifugation and a processing time (35–40 min), including plasma separation.

An alternative automated microfluidic-based plasma separation approach that involves an inertial separation structure has been reported (Zhou et al., 2020). Fundamental to the plasma collection reliability of this device is the flowrate at which whole blood was propelled through the inertial separation channels. Whole blood was actuated using a peristaltic pump which might not be entirely suited to POCT as cross-contamination could occur between assays if appropriate measures such as internal washing using pre-loaded buffers between assays or intermediary liquids to propel whole blood samples for example, are not employed. Therefore, if this approach was adapted for POCT, either on-chip micropumps or features such as diaphragms controlled by the system analyser may be required. Nevertheless, following plasma separation, this platform isolated exosomes using an antibody that recognised a specific tetraspanin and introduced an HRP-labelled detection antibody to quantify the concentration of bound EVs from small sample volumes.

Several of the platforms excluded pre-treatment methods, with one group developing an acousto-fluidic device to isolate exosomes directly from saliva by generating standing surface acoustic waves (sSAW) (Wang et al., 2020). Surface acoustic wave (SAW) technologies are frequently employed to control and manipulate particles in fluids and are particularly fitting for microfluidic devices. The sSAW method utilised two pairs of interdigital electrode transducers, the first of which forced larger particles (micrometre) towards a waste channel and the second pair removed remaining sub-micrometre particles larger than exosomes (>150 µm). This device demonstrated superior exosome yields in comparison with differential centrifugation and the extracted exosomes were consequently processed for the analysis of viral DNA using a droplet digital PCR assay performed on an external system.

**FIGURE 6:**
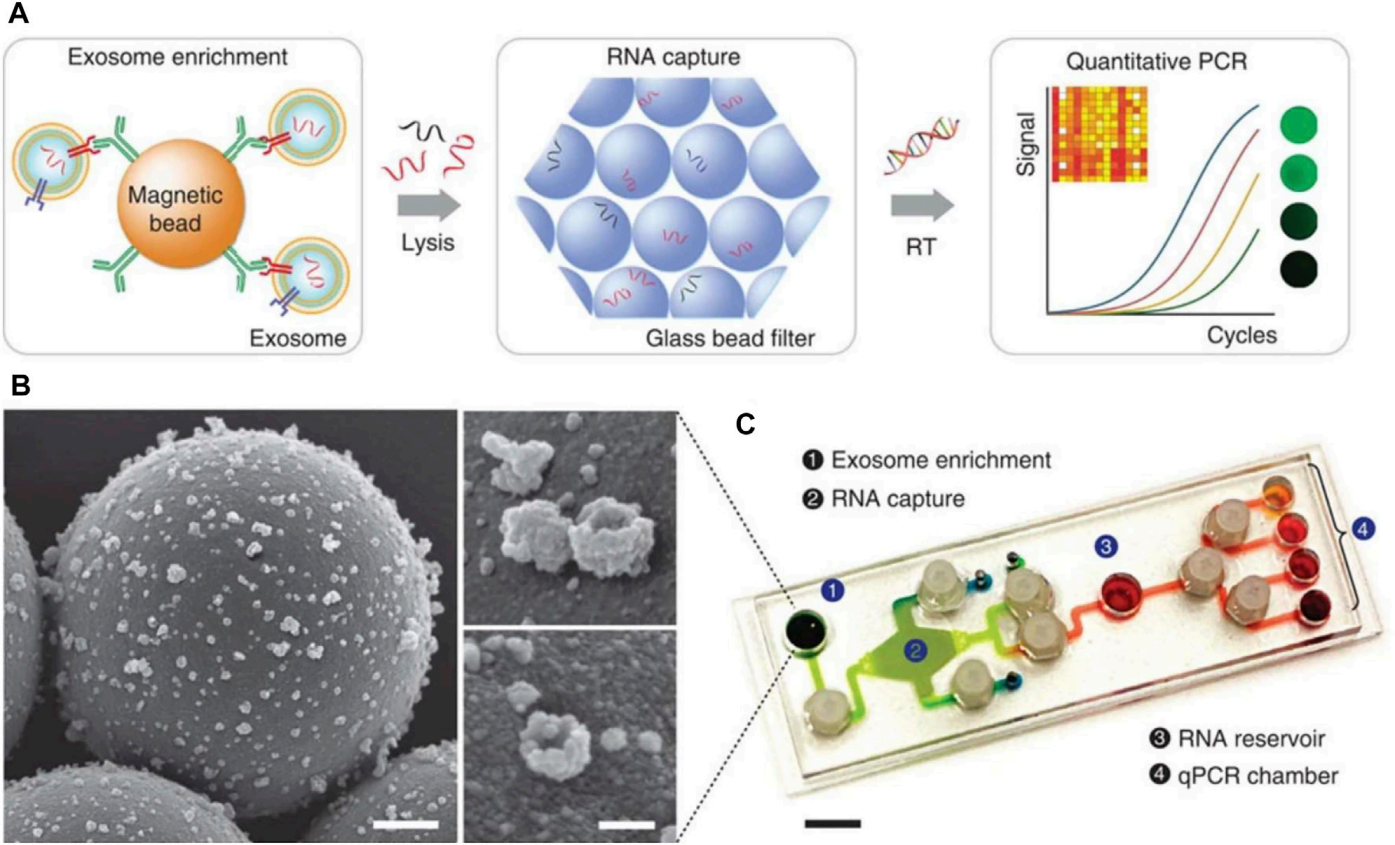
Diagram of key steps/approaches for measuring exosome for the prediction of CVD. **(A)** Magnetic microbeads coated with specific antibodies enabled exosome isolation and addition of guanidine-based lysis buffer released the contents of enriched exosomes. Torque-actuated valves and permanent magnets were used to control the process. RNA is adsorbed on a glass-bead filter *via* electrostatic interactions and reagents are loaded to prepare for qPCR. **(B)** Images from scanning electron microscope of exosomes captured on antibody-coated magnetic microbeads. Scale bars 500 and 100 nm (inset) **(C)** Photograph of PDMS iMER cartridge. RT—Reverse transcription (Shao et al., 2015). Source: Reprinted in its original form under CC BY license. License access: https://creativecommons.org/licenses/by/4.0/legalcode.

Other groups report the recovery of sample EVs using tailored microfluidic-based devices, such as the nPLEX (Im et al., 2014) and iMER (Shao et al., 2015) platforms, which also support on-chip biochemical analysis. The basic operation principle of the iMER platform involves the recovery of sample exosomes, on-chip lysis, RNA enrichment and subsequent analysis of captured RNA. A complementary customised PCR system, including a thermal cycler and a fluorescence detector, facilitated RNA detection (Figure 6). The iMER platform required approximately 100 µl serum to measure exosome-derived RNA within 2 h; although this is a substantial improvement compared to laboratory-based techniques, to warrant adaption for POCT, the TAT would have to be reduced. Hence, this approach is possibly more suited to high-throughput laboratory analysis.

It must be emphasised that embedding multiple stepwise operations within a microfluidic device to isolate exosomes and measure their contents requires extended periods of time to accommodate the analytical process. Combining some of the more rapid exosome enrichment microfluidic-based techniques with emerging accelerated PCR assays may prove to support shorter TATs and permit integration within rapid sample-to-answer POC platforms (Bustin, 2017). Although, this would require further investigation to establish the exact configuration of a suitable exosome enrichment POC assay, its economic feasibility and justification of its development for a particular application. Conversely, exosome-derived RNA can be measured without employing a time-consuming exosome isolation process using a bespoke SPR-based exosome lysis platform (Ramshani et al., 2019). Reverse transcription PCR (RT-qPCR) was conducted to measure free-floating RNA in plasma samples. Remaining plasma was subjected to SAW to lyse sample exosomes and prepare for further RNA measurement. The difference between the RNA concentration before and after the lysis of the exosomes could be attributed to the release of EV-bound RNA. This complete approach required 1 h, with 30 min allocated for each RT-qPCR assay. If this approach was performed simultaneously, i.e., free-floating and lysed total RNA measured concurrently, in addition to adopting a rapid PCR assay (Chan et al., 2016; Eboigbodin et al., 2017), this method could enable rapid measurement of exosome-derived RNA. Crucially, this strategy may not be suitable for certain exosome-derived biomarkers, specifically proteins. However fundamentally, it illustrates the premise by which exosome-derived RNA could be measured in a manner that is fitting for POCT.

## Conclusion

It is well established that endothelial dysfunction is a key atherosclerotic mechanism that without the balanced presence of corresponding athero-protective factors, leads to intimal thickening and progression of arteriosclerotic disease. Currently, cIMT is regarded as one of the primary surrogate indices of atherosclerosis. However, in some AMI patients, interrogation by angiography reveals no significant lesions within surveyed coronary beds. Fundamental to individual risk profiling and the diagnosis of ACS is the measurement of biomarkers, typically from collected whole blood specimens. Endothelial exosomes found in liquid biopsies may present as ideal cardiac biomarkers because they are involved in the progression of atherosclerosis and enhanced secretion has been demonstrated during periods of cellular stress.

Performing liquid biopsies is a potentially minimally-invasive route to elucidating underlying functional disturbances; however, the prospect of exploiting exosomes as diagnostic biomarkers of CVD is intrinsically dependent on the efficiency at which they can be acquired for analysis. Currently, isolating exosomes requires laborious processes that are conducted in laboratory settings and exhibit differing capabilities in attaining consistent yields and extract purity. Many of these approaches offer suboptimal performances and are inherently unsuited to POCT or efficient high-throughput measurements. Hence, tailored microfluidic-based devices favour adaption of exosomal analysis for diagnostic platforms and provide greatly reduced processing times. These devices—some of which incorporate automated pre-treatment—can efficiently enrich exosomes from whole blood, perform lysis and supply a nucleic acid, protein and lipid rich extract for subsequent analysis. The significance of exosomes and the cargo they harbour remains in its infancy, yet it is anticipated that greater detail will be revealed regarding their role in CVD and how best to interpret their fluctuating presence during disease progression. Ultimately, their clinical value for predicting CVD will be defined by a combination of diagnostic insight which they afford, therapeutic strategies which they permit and the ease at which they can be reliably and consistently isolated from samples.
